# ﻿Nomenclature and taxonomic identities of *Prunuszappeyana* and P.zappeyanavar.subsimplex (Rosaceae)

**DOI:** 10.3897/phytokeys.190.80490

**Published:** 2022-02-17

**Authors:** Bao-Huan Wu, Da-Fang Cui, Ming Kang

**Affiliations:** 1 Key Laboratory of Plant Resources Conservation and Sustainable Utilization, South China Botanical Garden, Chinese Academy of Sciences, Guangzhou 510650, China; 2 College of Forestry and Landscape Architecture, South China Agricultural University, Guangzhou 510642, China

**Keywords:** *Cerasus*, China, taxonomy, typification

## Abstract

The original specimens of both *Prunuszappeyana* and P.zappeyanavar.subsimplex were found to belong to more than one taxon. In addition, P.zappeyanavar.subsimplex was found to be invalid because, when the name was published, two separate descriptions were given to two cited collections, but not to the taxon, making the name unaccompanied with a description or diagnosis of this taxon (Art. 38.1 (a)). Therefore, a lectotype of *P.zappeyana* was designated under Art. 9.11 of ICN, by which *P.zappeyana* was placed in the synonymy of *P.veitchii*.

## ﻿Introduction

Prunussubg.Cerasus (Mill.) A. Gray is a taxonomically complex group, commonly known as cherries (Liu et al. 2018). There are 39 species or varieties of China recognised in ‘*Flora of China*’ (Li and Bartholomew 2003), whose taxonomy remains partly unresolved. A recent study found that original materials containing taxonomically discordant elements may be responsible for some taxonomic confusion in this subgenus (Wu et al. 2019). In our recent work, we found another example of this kind, which we clarify below.

In 1912, Koehne (1912) described *P.zappeyana* Koehne, based on *Wilson 45* and *Wilson 70* (part). In the same work, he established a new variety named P.zappeyanavar.subsimplex Koehne, based on *Wilson 45a* and *Wilson 3526*. After the publication of these two taxa, Schneider (1912) recognised *P.zappeyana*, but mentioned P.zappeyanavar.subsimplex as uncertain in his famous dendrological encyclopaedia ‘*Illustriertes Handbuch der Laubholzkunde*’. Thereafter, with no explanation, Silva Tarouca and Schneider (1922) indicated *P.zappeyana* to be a synonym of *P.concinna* Koehne (which was treated as a synonym of *P.veitchii* Koehne by Wu et al. (2019)) by placing the former in parentheses following the latter. After that, these two taxa were neglected until Li and Bartholomew (2003) unexplainably synonymised them with *P.trichostoma* Koehne, which is totally different from the treatment of Silva Tarouca and Schneider (1922).

To resolve the incongruence between these two taxonomic treatments, in this study, we investigated the nomenclature and the identities of *P.zappeyana* and P.zappeyanavar.subsimplex with the reference to their protologues and original materials.

## ﻿Results and discussion

In the protologue of *P.zappeyana*, Koehne (1912) expressed his concern about the identities of *Wilson 45* and *Wilson 70*, saying that the leaves of the former are larger, while the leaves of the latter are smaller. Similarly, he was somewhat doubtful about the identities of *Wilson 45a* and *Wilson 3526*. He did not combine the morphological descriptions of the two collections into a single description as he did when describing other taxa, but gave two descriptions of the two collections separately.

Our examination of the original collections confirmed Koehne’s concern on the identities of the original collections, demonstrating that both the original specimens of *P.zappeyana* and P.zappeyanavar.subsimplex represent at least two different taxa.

**Figure 1.**
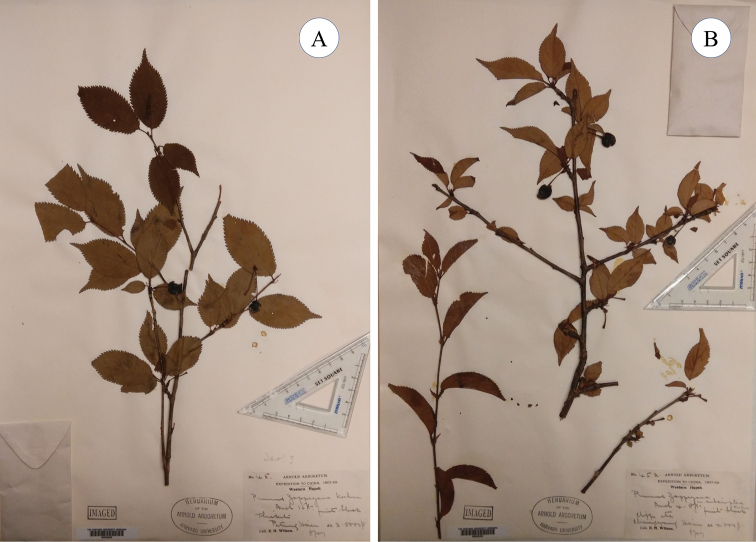
Original specimens of *Prunuszappeyana* and P.zappeyanavar.subsimplex**A** lectotype of *P.zappeyana*, *Wilson 45* (A 00032250) **B** one of the original specimens of P.zappeyanavar.subsimplex, *Wilson 45a* (A 00032252).

For *P.zappeyana*, we have successfully sorted out one sheet of *Wilson 45* kept at A and two sheets of *Wilson 70* kept at E and US. The specimen of *Wilson 45* (A 00032250, Fig. 1A) consists of a leafy branch with mature fruits and a small leafy branch and obviously belongs to *P.veitchii* Koehne. The leaves on this specimen are obovate-elliptic, 3.5–6.5 cm long, 1.8–3 cm broad, with netted veins that are prominent on the leaf back, with leaf margins serrate or biserrate and petioles 0.5–0.8 cm long. The peduncles are sessile. The fruits are ovoid to nearly globular and black.

In the protologue, Koehne (1912) indicated that *Wilson 70* partly belongs to *P.zappeyana* and partly to P.pilosiusculavar.barbata Koehne. We successfully traced two sheets of *Wilson 70* (E 00011305 and US 03718362) kept at E and US, both of which were determined as *P.zappeyana* by Koehne. However, we have failed to locate any specimen of *Wilson 70* that was annotated as P.pilosiusculavar.barbata. The specimen of *Wilson 70*, housed at E, was determined by T.-T. Yu in July 1948 as *P.latidentata* Koehne (which was reduced as a synonym of *P.trichostoma* in ‘*Flora Reipublicae Popularis Sinicae*’ (Yu and Li 1986)) and then was annotated by C.-L. Li in 1994 as an isotype of *P.zappeyana*, which was subsequently corrected to a syntype by herbarium curators. It is reasonable to infer that *P.zappeyana* was synonymised by Li and Bartholomew (2003), based on the specimen of *Wilson 70* kept at E. Regarding the identification of these two specimens of *Wilson 70*, we agree that they can be identified as *P.trichostoma* s.l., but we think they can also be determined as *P.stipulacea* Maxim. which is distinguished from *P.trichostoma* by blossoming slightly before the leaves (or nearly so) and ovate or auriculate stipules on vegetative branches (Li and Bartholomew 2003). Both specimens of *Wilson 70*, kept at E and US, only carry leafy branches with short branchlets and lack the important diagnostic characters.

For P.zappeyanavar.subsimplex, we located one sheet each of *Wilson 45a* (A 00032252, Fig. 1B) and *Wilson 3526* (A 00032251) kept at A and E. The specimen of *Wilson 45a* carries two leafy branches with infructescences and a young leafy branch. This specimen should also be identified as *P.veitchii*. The plants on this specimen show features similar to *Wilson 45*, such as the obovate-elliptic leaves with serrate or biserrate leaf teeth, sessile peduncles and ovoid to subglobular fruits in black colour. The specimen of *Wilson 3526* contains two branches with flower buds that are mostly not open and leaves that are not expanded. This specimen could be identified as *P.clarofolia* Schneider. The leaves of this specimen are mostly simply serrate and toothed with minute apical glands. The leaf back is pubescent along veins and the petioles are glabrous. The inflorescences are umbellate with 1–2 flowers, with bracts that are toothed with capitate apical glands. The pedicels and hypanthium are glabrous.

According to Art. 9.11 of ICN (Turland et al. 2018), it is necessary to designate the lectotypes of *P.zappeyana* and P.zappeyanavar.subsimplex. However, P.zappeyanavar.subsimplex is invalid because, when Koehne (1912) established P.zappeyanavar.subsimplex, two descriptions were separately given to the collections, but no combined description under the name directly, making the name unaccompanied by a description of this taxon (Art. 38.1(a)). Therefore, only the lectotypification of *P.zappeyana* is proposed here.

Either *Wilson 45* or *Wilson 70* can be chosen as the lectotype of *P.zappeyana* as they both correspond to the original description and match the diagnosis. We prefer to choose *Wilson 45* over *Wilson 70* as the lectotype of *P.zappeyana* for two reasons. First, as the identification of *Wilson 70* remains taxonomically ambiguous, we prefer to choose *Wilson 45* to make the identity of *P.zappeyana* more unambiguous. Second, we think Koehne apparently considered *P.zappeyana* and P.zappeyanavar.subsimplex as belonging to the same species; and *Wilson 45* and *Wilson 45a* are the nomenclatural elements which provide a common taxonomy to link these two names together, though the latter was not validly published.

Therefore, we chose the specimen of *Wilson 45* as the lectotype of *P.zappeyana* and, furthermore, treated *P.zappeyana* as the synonym of *P.veitchii*, based on the lectotype. This decision deviates from the taxonomy of Li and Bartholomew (2003), but agrees with the older interpretation by Silva Tarouca and Schneider (1922). It makes no changes to the currently accepted nomenclature of Chinese cherries, but contributes to its further disambiguation.

## ﻿Taxonomic treatment

### ﻿ Prunus veitchii

Taxon classificationPlantaeRosalesRosaceae

Koehne, Pl. Wilson. (Sargent) 1(2): 257. 1912.

Prunus veitchii Type: China. Western Hubei, April 1900, *E.H. Wilson 66* (lectotype designated by Wu et al. (2019: 66), US00130697, image!; isolectotypes E00417568, image!, HBG511147, image! NY00415930, image! A00032230 part, image!)
= Prunuszappeyana Koehne, Pl. Wilson. (Sargent) 1(2): 221. 1912, syn. nov. Type: CHINA. Hubei Province: Badong (Patung) County, alt. 1000–1600 m, June 1907, *E.H. Wilson 45* (lectotype designated here: A00032250!) (Fig. 1A). Remaining syntype: China. Hubei, woods, alt. 1300–2000 m, June 1907, *E. H. Wilson 70* (E 00011305, image!).

#### Note.

For a full list of synonyms, descriptions and distribution of *Prunusveitchii*, see Wu et al. (2019).

## Supplementary Material

XML Treatment for Prunus veitchii
